# Opium

**Published:** 1852-06

**Authors:** 


From the New London Pharmacopœia.

*Opium.*

OPIUM may be used to allay the irritability caused by excessive loss of blood. In the passage of gall stones, or of renal calculi, it is imperatively called for to allay the excruciating pain, and by relaxing the biliary duct or the ureters, to favor the passage of the stone. In the phosphatic diathesis, it is the only thing which has much power in allaying the morbid irritability of the system (Prout). In chordee. In diabetes it has proved more valuable than any other medicine which has been employed; it must be given in frequent and full doses. In poisoning by vegetable acids it is useful in allaying pain and moderating the violence of their action; and it is often required to relieve the symptoms which occur at a later period in poisoning by the mineral acids. In mortification, if accompanied by much pain, and in gangrena senilis, Mr. Pott's strong recommendations of opium have been fully confirmed by subsequent experience. In phagedenic ulceration it is all important, and must be given largely to allay the intense pain. In many callous or varicose ulcers, in old people whose health is enfeebled by sickness or intemperance, the use of small doses of laudanum (min. x., ter quotidie) produces great improvement; "it appears to promote the most genial warmth, to give energy to the extreme arteries, and thereby maintain an equal balance to the circulation, throughout every part of the body;" and to animate the dormant energies into healthy action.

The employment of opium in the constitutional treatment of severe surgical injuries is very little, if at all noticed by writers on Materia Medica; but it is most important. In many cases, the shock to the system and the subsequent irritation which is set up, are so great, especially if the injury is considerable amongst tendinous parts, that large quantities of this drug are required to tranquilize the system. The following case in the Toxteth Hospital, under the care of Mr. Minshull, of this town, [Liverpool] was communicated to me by Mr. Metcalf, then resident surgeon, and illustrates the general principles of its employment. A ship's baker of confirmed intemperate habits, who was invariably drunk once a week or oftener, and sometimes continued so for two or three days, fell, whilst intoxicated, and produced a compound dislocation of the left, and a simple dislocation of the right knee. There was great reason to believe that he had long practiced onanism, and he was, in every respect, a most unfavorable subject. Amputation in the middle of the thigh was immediately performed, and the right knee was reduced. In about two hours after the operation, delirium tremens began to show itself, and f℥ij of laudanum were given at once; he became tranquilized, and the dose was repeated in two hours, and again in three hours; after which, f℥j was given every two or three hours during the first twenty-four hours; the only effect produced was tranquility; he did not sleep, but was free from pain; the pulse was soft and steady, at about 70; the tongue moist, skin soft, and no thirst. The second day one or two grains of opium were given every two or three hours, according to his condition: if there were any symptoms of restlessness, the large dose was given, and it was more quickly repeated. On the third day, one grain was given, at longer intervals; he scarcely slept during this time, but was perfectly easy; he had little desire for food and no thirst, and the stump healed favorably. In six weeks he was walking about, and in good health. It very frequently happens that, if a patient has been of at all dissipated habits, or lives in a crowded or unhealthy situation, a wound resulting from an injury takes on a painful or unhealthy action; in this case the best treatment is frequently to give opium freely; local applications, or depletion, as by purgatives, either do no good, or aggravate the mischief.

Locally, opium is employed, in the form of plaster or liniment, in various cases of local pain, as bruises or chronic rheumatism. In neuralgia it is not nearly so efficacious as belladonna or aconite. In painful ulcers an opium lotion is frequently an admirable application. In ophthalmia, the vinum opii is dropped into the eye, and allays the pain and irritability, whilst it acts as a stimulant to the congested vessels. In spasmodic stricture and chordee, and in diseases of the prostate gland, and in dysentery, an opium suppository is often useful. In diarrhœa, and some other diseases in which it may be advisable not to give it by mouth, it is advantageously administered in an enema. The French consider opium to produce more powerful effects in this way than when taken by the mouth; but English experience does not confirm this, and the dose thus given is usually double the ordinary one. Care must be taken that the quantity of fluid in the enema is not too large; two or three ounces, slowly injected, is sufficient; if more than this is used, it will probably be returned.

*Dose and Administration.*—Small dose, gr. ⅛ to gr. ss. Medium, gr. ss. to gr. j or gr. jss. Full, gr. ij to gr. v; but much larger doses may sometimes be given. Of the tincture, min. ij to fʒj or fʒij. The tincture contains about one grain of opium in nineteen minims; hence min. ij correspond nearly with gr. 1-10th of solid opium. By enema about min. xxx is a medium dose. As a suppository, gr. v may be mixed with soap. As has been already mentioned, the dose for infants should be exceedingly small, and most carefully watched; under two months of age ⅛ of a drop is as much as is safe, and one drop is a full dose for a child a year old. When combined with calomel, it restrains the action of this medicine upon the bowels, and more speedily induces its constitutional effects. Combined with ipecacuanha, it checks its nauseating effects, causes diaphoresis, and has its own effects upon the head diminished in a remarkable degree; similar effects are produced by its combination with tartar-emetic.

